# Feasibility of Ultrasound-Guided Trigger Point Injection in Patients with Myofascial Pain Syndrome

**DOI:** 10.3390/healthcare7040118

**Published:** 2019-10-15

**Authors:** Jung Joong Kang, Jungin Kim, Seunghun Park, Sungwoo Paek, Tae Hee Kim, Dong Kyu Kim

**Affiliations:** Department of Rehabilitation Medicine, Konkuk University Chungju Hospital, Konkuk University School of Medicine, Chungju 27376, Korea; etk1025@gmail.com (J.J.K.); jsm00339@naver.com (J.K.); silveryluna@gmail.com (S.P.); ys97157@naver.com (S.P.); whitepoem37@naver.com (T.H.K.)

**Keywords:** shoulder, superficial back muscles, myofascial pain syndromes, trigger points, ultrasonography

## Abstract

We compared the feasibility of ultrasound (US)-guided myofascial trigger point (MTrP) injection with that of a blind injection technique following the use of shear wave elastography (SWE) for the measurement of stiffness at the MTrPs in patients with trapezius myofascial pain syndrome (MPS). A total of 41 patients (*n* = 41) were randomized to either the trial group (*n* = 21, SWE combined with US-guided injection) or the control group (*n* = 20, SWE combined with blind injection). At baseline and four weeks, they were evaluated for the manual muscle test (MMT), the range of motion (ROM), pain visual analogue scale (VAS) scores, Shoulder Pain and Disability Index (SPADI) scores and Neck Disability Index (NDI) scores during the abduction, adduction, flexion, extension, external rotation and internal rotation of the shoulder joint. Differences in changes in pain VAS scores, NDI scores and SPADI scores at four weeks from baseline between the two groups reached statistical significance (*p* = 0.003, 0.012, and 0.018, respectively). US-guided MTrP injection is a more useful modality as compared with a blind injection in patients with MPS.

## 1. Introduction

Myofascial pain syndrome (MPS) is referred to as regional pain that arises from hyperirritable spots located within taut bands of the skeletal muscle, termed as myofascial trigger points (MTrPs). Its common etiologic factors include direct or indirect trauma, spine pathology, exposure to cumulative and repetitive strain, postural dysfunction and physical de-conditioning. Current treatment strategies for MPS are the elimination of underlying etiologic factors. Unless appropriately treated, such etiologic factors would reactivate MTrPs and then cause a persistent presence of MPS [1,2,3,4].

According to Simons, MTrPs are a commonly overlooked source of muskuloskeletal disorders although they commonly cause pain in patients with MPS. They are identified at an estimated prevalence of 30–93% in patients complaining of musculoskeletal pain. Indeed, there is great variability in the overall prevalence of active MTrPs, estimated at 46.1 ± 27.4% [5,6,7]. Presumably, this might be because there is still a lack of definite consensus on criteria for defining MPS although it is a curable entity [8,9].

MTrPs are often present in the upper trapezius muscle, which accounts for approximately 85% of patients with MPS presenting with pain [10]. They can be either active or latent, active ones cause spontaneous pain and motor symptoms with nerve stimulation, whereas latent ones do not cause painful symptoms. Moreover, biochemical mediators, such as bradykinin and serotonin, are present in active MTrPs [11]. For the treatment of MTrPs, pharmacological agents, manual and physical therapy, dry needling, MTrPs injection, and botulinum toxin injections have been used [12].

With technological advancements in imaging modalities, such as ultrasound (US), magnetic resonance imaging (MRI) or positron emission tomography (PET), it has become possible to make an accurate identification of biomarkers with a predictive value [13,14]. Thus, attempts have been made to diagnose peripheral neuropathy and local myopathy and to perform dry needling at MTrPs using a US [13,15]. This has led to a new approach to US-guided treatment of MTrPs, thus showing that it was effective in significantly not only raising the accuracy and specificity of detection of MTrPs and but also achieving treatment effects [13].

Over the past decade, new in vivo imaging modalities, such as magnetic resonance elastography and US elastography, have emerged as a quantitative method for measuring shear mechanical properties of the soft tissue (e.g., the velocity of propagation of shear wave), including the skeletal muscle, through external induction of the shear wave or with the use of radiation force of US. Since then, its usefulness has been extended for a variety of tissues, such as liver, breast, biceps, trapezius, and rectus femoris muscle [16,17]. Considerable efforts have also been made to characterize the neuromuscular activation in patients with MPS. Lin et al. used a multi-scale wavelet model for the interpretation of surface electromyography (SEMG) signals, thus attempting to validate its usefulness in characterizing changes in neuromuscular activation in patients with MPS via machine learning [18]. Moreover, Jiang et al. advocated the use of a 3-dimensional multi-scale wavelet energy variation graph to analyze the SEMG variation between the dominant and nondominant sides at varying frequency scales during a muscle contraction cycle and the associated changes with the upper-back myofascial pain [19].

Given the above background, we conducted this preliminary pilot study to compare the feasibility of US-guided MTrP injection with that of a blind injection technique following the use of shear wave elastography (SWE) for the assessment of stiffness at the MTrPs in patients with MPS.

## 2. Materials and Methods

### 2.1. Study Patients and Setting

Between 1 April and 31 May 2019, a total of 41 patients (*n* = 41) were diagnosed with MPS at the department of rehabilitation of our medical institution and then evaluated in the current study.

Inclusion criteria for the current study are as follows:

(1) The patients aged between 40 and 49 years
(2) The patients with active MTrPs presenting with spontaneous pain and tender nodules
(3) The patients with a more than 1-month history of a palpable tender nodule in the upper trapezius [20].

Exclusion criteria for the current study are as follows:

(1) The patients presenting with symptoms that are similar to the MPS (e.g., cervical radiculopathy due to structural abnormalities or injuries to muscles or tendons in the shoulder and neck) [20].
(2) The patients with serious underlying diseases that may cause tissue degeneration (e.g., diabetes mellitus)
(3) The patients with cerebrovascular disorders that may cause spasticity in the morbid muscle (e.g., non-focal cerebral hemorrhage or cerebral infarction)
(4) The patients with central nervous system injuries (e.g., spinal cord injury)
(5) The patients who were currently taking muscle relaxants or physical therapy
(6) The patients who received a dry needling
(7) The patients who are deemed to be ineligible for study participation according to our judgment (e.g., those with psychiatric disorders).

We, therefore, enrolled a total of 41 patients (*n* = 41) in the current study, it was approved by the Institutional Review Board (IRB) of our medical institution (IRB approval # KUCH2019-04-012). The patients submitted a written informed consent. All procedures performed in this study were in accordance with the 1964 Declaration of Helsinki and its later amendments or comparable ethical standards.

### 2.2. Patient Evaluation and Criteria

**Figure 1:**
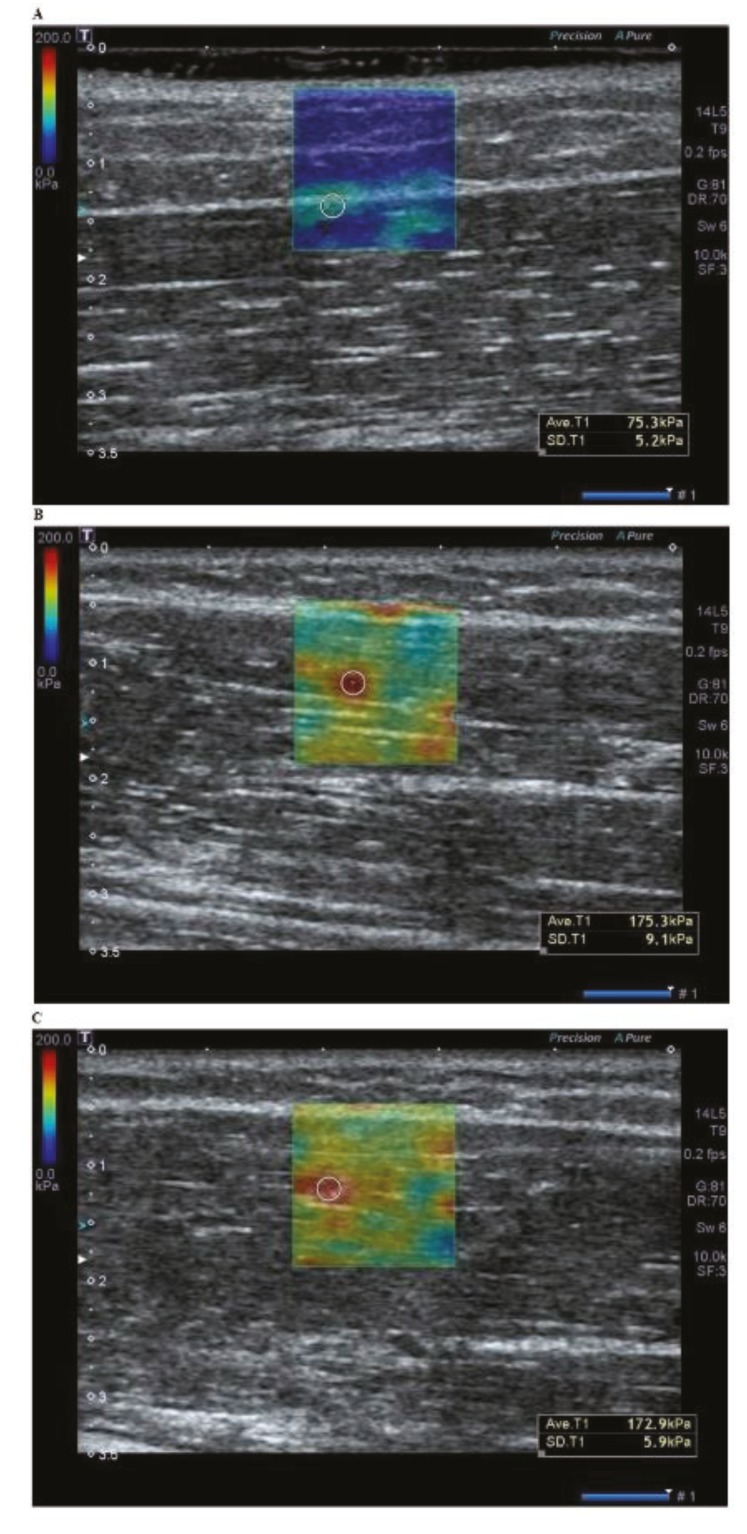
A shear wave elastography of the trapezius muscle at baseline. (**A**) No increased stiffness is seen in the asymptomatic muscle. (**B**) Increased stiffness at the myofascial trigger point is seen within the region of interest (ROI) in the posterior neck. (**C**) Increased stiffness is seen as a band form, not at the single location within the ROI.

At baseline, at the outpatient clinic on interview and physical examination, the patients were evaluated for the manual muscle test (MMT) based on the Medical Research Council (MRC) grading system, the range of motion (ROM), pain VAS scores, the Shoulder Pain and Disability Index (SPADI) scores and the Neck Disability Index (NDI) scores during the abduction, adduction, flexion, extension, external rotation and internal rotation of the shoulder joint, the daily use of analgesics and the number of palpable tender nodules. Then, they were also evaluated for the measurement of stiffness at the MTrPs on SWE using the Aplio 400 (Toshiba Medical System Co., Otawara, Tochigi, Japan) (Figure 1).

Using a permuted block design, the patients were randomized to either of the two treatment groups: the trial group (*n* = 21, US-guided injection combined with SWE) and the control group (*n* = 20, blind injection combined with SWE). In both groups, an SWE was performed in transverse imaging planes prior to injections, the tender point was located at the site where there was an increase in the stiffness at the MTrPs on SWE and tenderness was palpated on physical examination by a physical medicine and rehabilitation physician. Thus, the degree of stiffness at the MTrP was confirmed as the increased shear wave speed (SWS) within the region of interest (ROI) in patients with MPS. The size of the ROI on SWE was set at 1.4 cm × 1.4 cm. In addition, the degree of stiffness at the MTrPs on SWE was measured within a range of 0-200 kPA (Figure 1).

**Figure 2:**
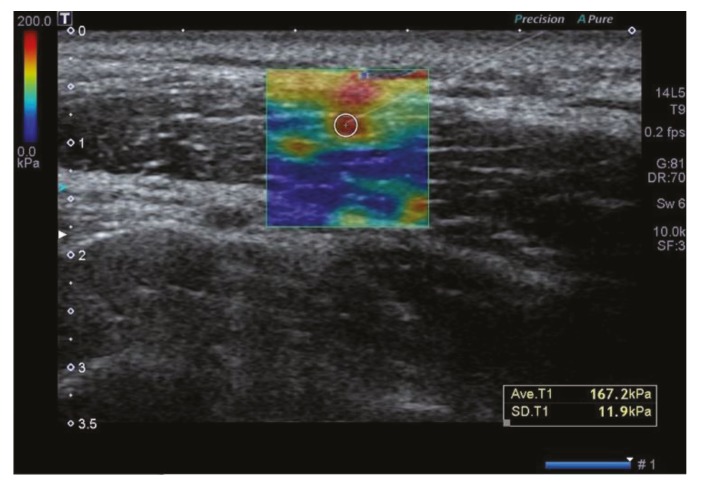
Ultrasound-guided myofascial trigger point (MTrP) injection. Increased stiffness at the MTrPs on shear wave elastography is confirmed on shear wave elastography. The injection was followed at the point where the stiffness increased mostly in the region of interest.

In the trial group, for US-guided injection, we checked the presence of muscle fibers forming the upper trapezius, thus attempting to rule out the involvement of other muscles, such as supraspinatus. This is followed by a US-guided injection of 1% lidocaine diluted with saline using a 3-mL syringe with a 4-cm, 23-G needle at a dose of 0.2 mL/point, for which the patients were placed in a sitting position (Figure 2). US-guided MTrP injection was performed by a board-certified specialist in physical medicine and rehabilitation with more than 10 years’ experience with sonographic detection of lesions using the Aplio 400 (Toshiba Medical System Co) and a linear array probe (5–14 MHz, PLT-1005 BT, Toshiba Medical System Co.). In the control group, after the detection of a palpable tender nodule on physical examination followed by SWE assessing the stiffness at the MTrPs, injections of 1% lidocaine diluted with saline were performed at the same dose using a blind technique, as proposed by Travell and Simons [20]. 

The patients of each group were instructed to daily perform an upper trapezius stretch at home, during which they did not receive any medications and physical therapies. Moreover, they were also instructed to keep a sticker attached to the MTrPs. Two weeks thereafter, they visited the outpatient clinic again and were evaluated for the MMT based on the MRC grading system, the ROM, pain VAS scores, SPADI scores and NDI scores during the abduction, adduction, flexion, extension, external rotation and internal rotation of the shoulder joint, the daily use of analgesics and the number of palpable tender nodules.

Differences in changes in the MMT based on the MRC grading system, ROM, the pain VAS scores, SPADI scores and NDI scores at 4 weeks from baseline between the two groups served as efficacy outcome measures.

### 2.3. Evaluation Tools

(1) MMT: An indicator of the muscle strength, the MRC grading system uses a 5-point scale: 0 = “Paralysis”, 1 = “Sole presence of a trace or flicker of the muscle contraction”, 2 = “The possibility of muscle movement with elimination of the gravity”, 3 = “The possibility of muscle movement against the gravity”, 4 = “Decreased muscle strength with the possibility of muscle movement against resistance”, and 5 = “Normal strength” [21].

(2) ROM: The ROM is defined as the amount of motion that is available at a joint. It was measured, as previously described by Norkin and White, for which the anatomical position served as the starting position except for rotations in the transverse plane [22].

(3) VAS: The VAS is composed of a straight line with the endpoints indicating extreme limits (‘no pain at all’ and ‘pain as bad as it could be’). For the assessment of pain, the patients were asked to mark their pain level on the line between the two endpoints. Accordingly, their pain level was defined as the distance extending from the point indicating ‘no pain at all’ and the mark [23].

(4) SPADI: The patients responded to a self-administered questionnaire study based on the SPADI. The SPADI was developed to measure the shoulder pain and disability in an outpatient setting. It includes 13 items, 5 and 8 of which evaluate pain and disability, respectively. It was tested and then validated in a mixed group of male patients with shoulder pain presenting to ambulatory care. Then, it has been used in a primary care setting and a surgical setting where patients with rotator cuff disease, osteoarthritis rheumatoid arthritis, adhesive capsulitis, joint replacement surgery or shoulder symptoms are included [24]. In the current study, we used the Korean version of the SPADI whose reliability and validity have been well described in the literature [25].

(5) NDI: A 10-item questionnaire measuring patients’ self-reported neck pain-related disability, the NDI has been shown to have a high “test-retest” reliability. As compared with other types of pain and disability measures, it has been found to be more valid. It comprises questions about activities of daily living, such as personal care, lifting, reading, work, driving, sleeping, recreational activities, pain intensity, concentration, and headache, each of which is measured based on a 5-point scale (0 = “No disability” and 5 = “Disability”). We used the Korean version of the NDI [26].

### 2.4. Statistical Analysis of the Patient Data

All data were expressed as mean ± SD (SD: standard deviation). Statistical analysis was done using the SPSS ver. 24.0 for windows (SPSS Inc., Chicago, IL, USA). For efficacy assessment, efficacy outcome measures were analyzed using the Student’s *t*-test. A *p*-value of <0.05 was considered statistically significant.

## 3. Results

### 3.1. Baseline Characteristics of the Patients

**Figure 3:**
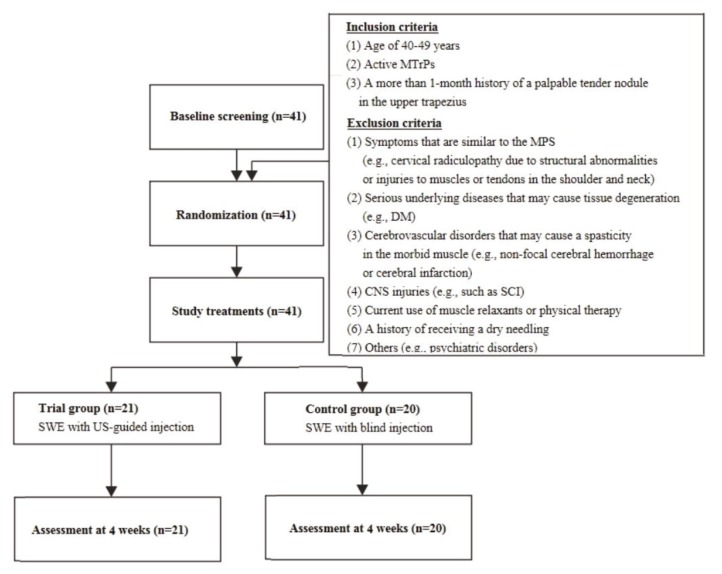
The study flow chart. MTrPs, myofascial trigger points. MPS, myofascial pain syndrome. DM, diabetes mellitus. CNS, central nervous system. SCI, spinal cord injury. SWE, shear wave elastography. US, ultrasound. The 41 eligible patients were randomized to either the trial group (*n* = 21) or the control group (*n* = 20), all of whom completed the current study.

A total of 41 patients (*n* = 41) with MPS were enrolled in the current study. They consist of 18 men and 23 women, whose mean age was 44.27 ± 2.22 years old. As shown in Figure 3, they were randomized to either the trial group (*n* = 21) or the control group (*n* = 20). Their baseline characteristics are represented in Table 1 and Table 2.

### 3.2. Efficacy Outcomes

As shown in Table 3, of efficacy outcome measures, differences in changes in pain VAS scores, NDI scores and SPADI scores at four weeks from baseline between the two groups reached statistical significance (*p* = 0.003, 0.012 and 0.018, respectively). These results indicate that the degree of efficacy was significantly higher in the trial group as compared with the control group (*p* < 0.05).

## 4. Discussion

Although unclear, pathophysiologic mechanisms underlying the formation of MTrPs in patients with MPS are explained by a cascade of inflammatory events. In more detail, levels of inflammatory mediators are elevated and this causes capillary compression resulting in tissue ischemia. With the increased metabolic demands, the muscle and its adjacent soft tissue undergo structural alterations and damages. The resulting persistent presence of sarcomere contracture is liable to a long-standing presence of myofascial tenderness and local pain at the MTrPs [27,28]. It has been therefore imperative that novel methods be identified for quantifying degeneration of the muscle tissue. Thus, musculoskeletal pathology was evaluated based on variability in the elasticity of soft tissue [29]. Moreover, a previous study reported a relationship between increased stiffness and pain [30]. This is also accompanied by another published study showing that active lengthening of the muscle fiber in the rigid tendon-aponeurosis complex caused a strain, which is supported by findings that effects of active lengthening are minimized by the tendon-aponeurosis complex and myofibrillar strain is inhibited accordingly. This is associated with shortening of the muscle fiber that may cause an increased stiffness [31,32,33].

Injections are frequently performed based on anatomical landmarks on palpation, thus termed as a blind technique [34]. It remains uncertain, however, whether there is an appropriate placement or misplacement of injected treatment agents at the MTrPs [35]. Thus, a blind technique would cause residual or earlier recurrence of symptoms [36]. To resolve this, US-guided injections of treatment agents have been attempted, whose accuracy has been well described in the literature [37].

The accuracy of US-guided injections of treatment agents is of significance, as previously described in a cadaver study, diagnostic or therapeutic US-guided injections are an extremely effective modality at diverse locations. US-guided injections should, therefore, be considered to maximize injection accuracy and minimize potential complications [37]. This is supported by previous studies about the increased accuracy of intra-articular injection leading to improved outcomes. US-guided injections showed a superior accuracy in identifying vital structures, such as nerves and vessels, and visualizing displacement of needle tip on a real-time basis as compared with fluoroscopy-guided ones [38,39].

To summarize, our results showed that differences in changes in pain VAS scores, NDI scores and SPADI scores at four weeks from baseline between the two groups reached statistical significance (*p* = 0.003, 0.012 and 0.018, respectively). These results indicate that the degree of efficacy was significantly higher in the trial group as compared with the control group (*p* < 0.05). However, limitations of the current study are as follows: First, we failed to serve treatment arms, such as the US-guided injection with or without SWE, for which valid outcome measures should be considered. Second, we evaluated only a small series of patients at the secondary medical institution located in a rural area. We could not therefore completely rule out the possibility of selection bias.

## 5. Conclusions

In conclusion, our results indicate that US-guided MTrP injection is a more useful modality as compared with a blind injection in patients with MPS. 

## Figures and Tables

**Table 1:** Baseline characteristics of the patients (*n* = 41).

Variables		Values
Age (years)		44.27 ± 2.22
Sex		
	Men	18
	Women	23
Daily use of analgesics		1.78 ± 0.80
ROM		
	Abduction	154.51 ± 7.40°
	Adduction	154.40 ± 6.34°
	Flexion	157.56 ± 6.14°
	Extension	64.36 ± 4.66°
	External rotation	92.44 ± 4.89°
	Internal rotation	96.34 ± 5.48°
MMT		
	Abduction	4.95 ± 0.22
	Adduction	4.95 ± 0.22
	Flexion	4.9 5 ± 0.22
	Extension	4.95 ± 0.22
	External rotation	4.93 ± 0.26
	Internal rotation	4.93 ± 0.26
Pain VAS scores		7.05 ± 0.62
NDI scores		20.54 ± 3.23
SPADI scores		38.32 ± 6.50
Number of palpable tender nodules		2.37 ± 0.80
Spasticity on SWE		159.63 ± 9.72

**Abbreviations:** MRC, Medical Research Council, ROM, range of motion, MMT, muscle manual test, VAS, visual analog scale, NDI, Neck Disability Index, SPADI, Shoulder Pain and Disability Index, SWE, shear wave elastography. Values are mean ± standard deviation or the number of cases with percentage, where appropriate.

**Table 2:** Baseline characteristics of the patients in each group.

Variables		Values		*p*-Value
		Trial Group (*n* = 21)	Control Group (*n* = 20)	
Age (years)		43.95 ± 2.18	44.60 ± 2.33	0.652
Sex				
	Men	9	10	
	Women	12	11	

Values are mean ± standard deviation or the number of cases with percentage, where appropriate.

**Table 3:** Efficacy outcomes.

Variables		Values						*p*-Value
		Trial Group (*n* = 21)			Control Group (*n* = 20)			
		0 week	4 weeks	Δ	0 week	4 weeks	Δ	
ROM								
	Abduction	154.52 ± 7.57°	155.00 ± 7.25°	0.48 ± 1.50°	154.50 ± 7.42°	154.50 ± 7.42°	0.00 ± 0.00°	0.162
	Adduction	154.40 ± 6.34°	154.76 ± 6.98°	0.00 ± 0.00	154.40 ± 6.34°	154.25 ± 5.45°	0.25 ± 1.12°	0.330
	Flexion	157.62 ± 6.25°	158.10 ± 5.80°	0.48 ± 1.50°	157.50 ± 6.18°	157.75 ± 5.73°	0.25 ± 1.12°	0.276
	Extension	64.52 ± 4.98°	64.76 ± 5.12°	0.24 ± 1.09°	64.75 ± 4.44°	65.00 ± 4.30°	0.25 ± 1.12°	0.945
	External rotation	92.14 ± 5.38°	92.38 ± 5.15°	0.24 ± 1.09°	92.75 ± 4.44°	93.00 ± 4.41°	0.25 ± 1.12°	0.945
	Internal rotation	96.67 ± 5.55°	96.90 ± 5.58°	0.24 ± 1.09°	96.00 ± 5.53°	96.25 ± 5.35°	0.25 ± 1.12°	0.945
MMT								
	Abduction	4.95 ± 0.22	5 ± 0	0.05 ± 0.22	4.95 ± 0.22	4.95 ± 0.22	0.00 ± 0.00	0.329
	Adduction	4.95 ± 0.22	4.95 ± 0.22	0.00 ± 0.00	4.95 ± 0.22	5.00 ± 0.00	0.05 ± 0.22	0.330
	Flexion	4.90 ± 0.30	4.95 ± 0.22	0.05 ± 0.22	4.95 ± 0.22	5.00 ± 0.00	0.05 ± 0.22	0.945
	Extension	4.95 ± 0.22	4.95 ± 0.22	0.00 ± 0.00	4.95 ± 0.22	5.00 ± 0.00	0.05 ± 0.22	0.330
	External rotation	4.95 ± 0.22	4.95 ± 0.22	0.00 ± 0.00	4.90 ± 0.31	4.90 ± 0.31	0.00 ± 0.00	0.207
	Internal rotation	4.90 ± 0.30	4.90 ± 0.30	0.00 ± 0.00	4.95 ± 0.22	4.95 ± 0.22	0.00 ± 0.00	0.276
Pain VAS scores		7.06 ± 0.61	5.14 ± 0.74	1.92 ± 0.56	7.03 ± 0.65	5.83 ± 1.24	1.20 ± 0.85	* 0.003
NDI scores		20.67 ± 3.28	9.52 ± 3.48	11.14 ± 4.19	20.40 ± 3.25	14.55 ± 7.32	5.85 ± 7.80	* 0.012
SPADI scores		38.67 ± 6.20	18.52 ± 8.86	20.14 ± 8.90	37.95 ± 6.95	28.25 ± 15.39	9.70 ± 16.39	* 0.018

**Abbreviations:** ROM, range of motion, MMT, muscle manual test, VAS, visual analog scale, NDI, Neck Disability Index, SPADI, Shoulder Pain and Disability Index, SWE, shear wave elastography. Values are mean±standard deviation or the number of cases with percentage, where appropriate. * Statistical significance at *p* < 0.05.

## References

[1] Leite F.M., Atallah Á.N., El Dib R., Grossmann E., Januzzi E., Andriolo R.B., da Silva E.M. (2009). Cyclobenzaprine for the treatment of myofascial pain in adults. Cochrane Database Syst. Rev..

[2] Desai M.J., Saini V., Saini S. (2013). Myofascial pain syndrome: A treatment review. Pain Ther..

[3] Chou L.W., Kao M.J., Lin J.G. (2012). Probable mechanisms of needling therapies for myofascial pain control. Evid. Based Complement. Altern. Med..

[4] Hong C.Z. (2006). Treatment of myofascial pain syndrome. Curr. Pain Headache Rep..

[5] Simons D.G. (2002). Understanding effective treatments of myofascial trigger points. J. Bodyw. Mov. Ther..

[6] Simons D.G. (1996). Clinical and etiological update of myofascial pain from trigger points. J. Musculoskelet. Pain.

[7] Fleckenstein J., Zaps D., Rüger L.J., Lehmeyer L., Freiberg F., Lang P.M., Irnich D. (2010). Discrepancy between prevalence and perceived effectiveness of treatment methods in myofascial pain syndrome: Results of a cross-sectional, nationwide survey. BMC Musculoskelet. Disord..

[8] Meyer H.P. (2002). Myofascial pain syndrome and its suggested role in the pathogenesis and treatment of fibromyalgia syndrome. Curr. Pain Headache Rep..

[9] Gerwin R.D. (2014). Diagnosis of myofascial pain syndrome. Phys. Med. Rehabil. Clin. N. Am..

[10] Yildirim M.A., Öneş K., Gökşenoğlu G. (2018). Effectiveness of Ultrasound Therapy on Myofascial Pain Syndrome of the Upper Trapezius: Randomized, Single-Blind, Placebo-Controlled Study. Arch. Rheumatol..

[11] Shah J.P., Phillips T.M., Danoff J.V., Gerber L.H. (2005). An in vivo microanalytical technique for measuring the local biochemical milieu of human skeletal muscle. J. Appl. Physiol..

[12] Narvani A.A., Tsiridis E., Kendall S., Chaudhuri R., Thomas P. (2003). A preliminary report on prevalence of acetabular labrum tears in sports patients with groin pain. Knee Surg. Sports Traumatol. Arthrosc..

[13] Bubnov R.V. (2012). Evidence-based pain management: Is the concept of integrative medicine applicable?. EPMA J..

[14] Condon B. (2011). Magnetic resonance imaging and spectroscopy: How useful is it for prediction and prognosis?. EPMA J..

[15] Chen Q., Bensamoun S., Basford J.R., Thompson J.M., An K.N. (2007). Identification and quantification of myofascial taut bands with magnetic resonance elastography. Arch. Phys. Med. Rehabil..

[16] Ballyns J.J., Turo D., Otto P., Shah J.P., Hammond J., Gebreab T., Gerber L.H., Sikdar S. (2012). Office-based elastographic technique for quantifying mechanical properties of skeletal muscle. J. Ultrasound Med..

[17] Nightingale K., Soo M.S., Nightingale R., Trahey G. (2002). Acoustic radiation force impulse imaging: In vivo demonstration of clinical feasibility. Ultrasound Med. Biol..

[18] Lin Y.C., Yu N.Y., Jiang C.F., Chang S.H. (2018). Characterizing the SEMG patterns with myofascial pain using a multi-scale wavelet model through machine learning approaches. J. Electromyogr. Kinesiol..

[19] Jiang C.F., Lin Y.C., Yu N.Y. (2013). Multi-scale surface electromyography modeling to identify changes in neuromuscular activation with myofascial pain. IEEE. Trans. Neural Syst. Rehabil. Eng..

[20] Shah J.P., Thaker N., Heimur J., Aredo J.V., Sikdar S., Gerber L. (2015). Myofascial Trigger Points Then and Now: A Historical and Scientific Perspective. PM R.

[21] Vanhoutte E.K., Faber C.G., Van Nes S.I., Jacobs B.C., Van Doorn P.A., Van Koningsveld R., Cornblath D.R., Van Der Kooi A.J., Cats E.A., Van Den Berg L.H. (2012). Modifying the Medical Research Council grading system through Rasch analyses. Brain.

[22] Wilk K.E., Reinold M.M., Macrina L.C., Porterfield R., Devine K.M., Suarez K., Andrews J.R. (2009). Glenohumeral internal rotation measurements differ depending on stabilization techniques. Sports Health.

[23] Haefeli M., Elfering A. (2006). Pain assessment. Eur. Spine J..

[24] Hill C.L., Lester S., Taylor A.W., Shanahan M.E., Gill T.K. (2011). Factor structure and validity of the shoulder pain and disability index in a population-based study of people with shoulder symptoms. BMC Musculoskelet. Disord..

[25] Seo H.D., Lee K.W., Chung K.S., Chung I.J. (2012). Reliability and Validity of the Korean version of Shoulder Pain and Disability Index. J. Spec. Educ. Rehabil. Sci..

[26] Song K.J., Choi B.W., Choi B.R., Seo G.B. (2010). Cross-cultural adaptation and validation of the Korean version of the neck disability index. Spine.

[27] Maher R.M., Hayes D.M., Shinohara M. (2013). Quantification of dry needling and posture effects on myofascial trigger points using ultrasound shear-wave elastography. Arch. Phys. Med. Rehabil..

[28] Vulfsons S., Ratmansky M., Kalichman L. (2012). Trigger point needling: Techniques and outcome. Curr. Pain Headache Rep..

[29] Lalitha P., Reddy M., Reddy K.J. (2011). Musculoskeletal applications of elastography: A pictorial essay of our initial experience. Korean J. Radiol..

[30] Jones D.A., Newham D.J., Clarkson P.M. (1987). Skeletal muscle stiffness and pain following eccentric exercise of the elbow flexors. Pain.

[31] McHugh M.P., Connolly D.A., Eston R.G., Kremenic I.J., Nicholas S.J., Gleim G.W. (1999). The role of passive muscle stiffness in symptoms of exercise-induced muscle damage. Am. J. Sports Med..

[32] Griffiths R.I. (1991). Shortening of muscle fibres during stretch of the active cat medial gastrocnemius muscle: The role of tendon compliance. J. Physiol..

[33] Shalabi N., Persson M., Månsson A., Vengallatore S., Rassier D.E. (2017). Sarcomere Stiffness during Stretching and Shortening of Rigor Skeletal Myofibrils. Biophys. J..

[34] So J.I., Song D.H., Park J.H., Choi E., Yoon J.Y., Yoo Y., Chung M.E. (2017). Accuracy of Ultrasound-Guided and Non-Ultrasound-Guided Botulinum Toxin Injection into Cadaver Salivary Glands. Ann. Rehabil. Med..

[35] Wong C.S., Wong S.H. (2012). A new look at trigger point injections. Anesthesiol. Res. Pract..

[36] Lamplot J.D., Lillegraven O., Brophy R.H. (2018). Outcomes from Conservative Treatment of Shoulder Idiopathic Adhesive Capsulitis and Factors Associated with Developing Contralateral Disease. Orthop. J. Sports Med..

[37] Muir J.J., Curtiss H.M., Hollman J., Smith J., Finnoff J.T. (2011). The accuracy of ultrasound-guided and palpation-guided peroneal tendon sheath injections. Am. J. Phys. Med. Rehabil..

[38] Daniels E.W., Cole D., Jacobs B., Phillips S.F. (2018). Existing Evidence on Ultrasound-Guided Injections in Sports Medicine. Orthop. J. Sports Med..

[39] Koh S.H., Lee S.C., Lee W.Y., Kim J., Park Y. (2019). Ultrasound-guided intra-articular injection of hyaluronic acid and ketorolac for osteoarthritis of the carpometacarpal joint of the thumb: A retrospective comparative study. Medicine.

